# A Ph.D. thesis entitled “Dosimetric studies on Hi-Tech Radiotherapy Systems and Sources and Development of Quality Assurance/ Audit Programmes”

**Published:** 2008

**Authors:** A. S. Pradhan

**Affiliations:** Former Head, Internal Dosimetry Division, Bhabha Atomic Research Centre, Trombay, Mumbai - 400 085, India. E-mail: ambika.s.pradhan@gmail.com

The above thesis by Sunil Dutt Sharma deals with the dosimetric aspects of beam therapy systems and brachytherapy sources **of** recent technology used for the treatment of cancer. It provides comprehensive dosimetric comparisons of different radiotherapy systems and sources and precise dosimetric methods and data for modern radiotherapy systems and sources which are of immense importance during clinical applications of these devices. The thesis also deals with the development of quality audit methods and programs for both beam and brachytherapy modalities which can be used for routine external audit of dosimetry data generated for patient dosimetry at radiotherapy centers.

Detailed experimental data of Siemens secondary multileaf collimator (MLC)–shaped high energy X-ray beams and a comparison with scatter factors of conventional jaw collimator (JC)–shaped X-ray beams of the same medical linear accelerator are presented. On the basis of these studies, it is demonstrated that the patient dosimetry formalism based on 10-cm reference depth gives better accuracy over depth-of-dose maximum formalism. The use of a suitable beam coaxial mini phantom for the measurement of collimator scatter factor is recommended to eliminate the unwanted contribution of contaminated electrons. Collimator and phantom scatter factors of high-energy X-ray beams are important parameters used for monitor unit calculations for patient treatment using a medical linear accelerator. The effect of contamination electrons and collimator exchange effects on the magnitude of these parameters are topics of current interest.

The advent and introduction of MLC in clinical practice has made it possible to adopt the technique of conformal radiotherapy. The method of shaping the conformal treatment portals depends on the type and technical aspects of the MLC. Accordingly, the degree of irregularity of the treatment field plays an important role in determining the magnitude of related dosimetry parameters. The author investigated the effect of degree of irregularity on the dosimetry parameters for conformal field shaped by Varian tertiary MLC and observed a significant change in the absolute output of the beam for the conformal field of very high degree of irregularity. This is an important observation which needs to be accounted for either mechanically or dosimetrically while using Varian tertiary MLC for conformal beam therapy. The quantification of percentage under dosing effect at the junction of MLC-shaped adjacent fields using radiochromic and radiographic films for the Varian tertiary MLC needs special mention as data presented is relevant to both conventional and intensity-modulated radiotherapy.

The steep dose gradient of very narrow photon beams used in stereotactic radiosurgery/radiotherapy (SRS/SRT) requires dosimeters with spatial resolution better than 1.0 mm. The lack of lateral electronic equilibrium further complicates the dosimetry. By using three different types of ionization chambers (PTW Markus plane parallel, PTW Pinpoint and Scanditronix CC01), an *in vivo* dosimetry diode (Victroreen VeriDose), micro cubes and powder grains of LiF:Mg, Ti thermoluminescence dosimeter (TLD), radiochromic film and radiographic film for dosimetry of X-Knife (Cone- as well as MLC-based) and Gamma Knife beams, the author studied the intricacies and made specific recommendations on the choice of dosimeter for small-field dosimetry. The work demonstrates that the practice of measuring output by PTW Markus plane parallel chamber and PTW Pinpoint chamber and using the average values of these two measurements is erroneous for SRS cone of diameter smaller than 12.5 mm.

Measured helmet output factors (HOF) of four different gamma knife units are also presented, indicating the unit-specific character of HOF.

The thesis also deals with dosimetry aspects of high–dose-rate (HDR) ^192^Ir source, indigenously developed low-energy photon-emitting ^125^I seed source and different gamma- and beta-emitting intravascular sources used for brachytherapy applications. Radiochromic film–based dosimetry methods are presented for in-water dosimetry of HDR ^192^Ir gamma ray source. It also demonstrates that radiochromic film can be used to generate dosimetry data at a high resolution suitable for comparison with the values obtained by Monte Carlo calculations. Accurate and precise methods using TLD micro rods and in-house fabricated PMMA phantoms are presented for generation of American Association of Physicists in Medicine (AAPM) TG-43–recommended dosimetry data of ^125^I seed source and for comparison with the Monte Carlo–calculated values. Simple dose rate tables presented for patient dosimetry while using ^125^I seed source for interstitial and ocular brachytherapy applications may be useful.

In the thesis, the author has also included the experimentally measured and Monte Carlo–calculated dosimetry features of ^32^P-coated stents, ^186^Re/^188^Re liquid–filled balloons, ^90^Sr/^90^Y seed trains and ^192^Ir seed ribbons used for intracoronary brachytherapy applications.

Quality audit (QAu) by mailed dosimeters is an alternative to fulfill partially the requirements stipulated under audit programs by various national/international agencies. TLD is a reliable, well-tested dosimeter and is in use from the last three decades for postal audit purposes. However, it is laborious and time consuming. Gafchromic EBT film–based comprehensive postal dosimetry method is demonstrated by the author for audit of important dosimetry parameters, both in reference and nonreference conditions of high-energy photon beams. The postal QAu method presented for measuring brachytherapy source strength and verifying dose to point ‘A’ and point ‘B’ delivered during intracavitary brachytherapy treatment also appears useful.

The work for Ph.D. degree carried out by S. D. Sharma at Radiological Physics & Advisory Division (RP&AD), Bhabha Atomic Research Centre (BARC), Mumbai, under the guidance of Dr. B. C. Bhatt (Former Head, RP&AD, BARC) (as a research guide) at the University of Mumbai, Mumbai, India, is of significant relevance to clinical dosimetry and quality audit of several parameters of radiotherapy systems and sources of modern technology used for the treatment of cancer patients.

